# Generation and Phenotyping of a Collection of sRNA Gene Deletion Mutants of the Haloarchaeon *Haloferax volcanii*

**DOI:** 10.1371/journal.pone.0090763

**Published:** 2014-03-17

**Authors:** Katharina Jaschinski, Julia Babski, Matthias Lehr, Anna Burmester, Juliane Benz, Ruth Heyer, Marcella Dörr, Anita Marchfelder, Jörg Soppa

**Affiliations:** 1 Goethe-University, Institute for Molecular Biosciences, Frankfurt, Germany; 2 Biology II, Ulm University, Ulm, Germany; State Key Laboratory of Pathogen and Biosecurity, Beijing Institute of Microbiology and Epidemiology, China

## Abstract

The haloarchaeon *Haloferax volcanii* was shown to contain 145 intergenic and 45 antisense sRNAs. In a comprehensive approach to unravel various biological roles of haloarchaeal sRNAs *in vivo*, 27 sRNA genes were selected and deletion mutants were generated. The phenotypes of these mutants were compared to that of the parent strain under ten different conditions, i.e. growth on four different carbon sources, growth at three different salt concentrations, and application of four different stress conditions. In addition, cell morphologies in exponential and stationary phase were observed. Furthermore, swarming of 17 mutants was analyzed. 24 of the 27 mutants exhibited a difference from the parent strain under at least one condition, revealing that haloarchaeal sRNAs are involved in metabolic regulation, growth under extreme conditions, regulation of morphology and behavior, and stress adaptation. Notably, 7 deletion mutants showed a gain of function phenotype, which has not yet been described for any other prokaryotic sRNA gene deletion mutant. Comparison of the transcriptomes of one sRNA gene deletion mutant and the parent strain led to the identification of differentially expressed genes. Genes for flagellins and chemotaxis were up-regulated in the mutant, in accordance with its gain of function swarming phenotype. While the deletion mutant analysis underscored that haloarchaeal sRNAs are involved in many biological functions, the degree of conservation is extremely low. Only 3 of the 27 genes are conserved in more than 10 haloarchaeal species. 22 of the 27 genes are confined to *H. volcanii*, indicating a fast evolution of haloarchaeal sRNA genes.

## Introduction

A few small non-coding regulatory RNAs (sRNAs or ncRNAs) have been known for many years, but were long thought to be exotic exceptions. This view has changed dramatically during the last decade and today it is clear that sRNAs are widespread in all three domains of life and have in fact been found in every species that has been tested [1]–[6]. The numbers of known sRNAs, their biological functions, and the different mechanisms of action that are uncovered, increase steadily.

In bacteria, sRNAs are 50 to 300 nucleotides in size and bacteria typically contain 100 to several hundred sRNAs. They are involved in stress response, virulence gene regulation, and in regulation of carbon source uptake and metabolism (for reviews see [2], [5]–[8]). Many sRNA genes are localized in intergenic regions and thus the sRNAs are encoded *in trans* to their target mRNAs. Typically, they function via imperfect base-pairing near the 5′-ends of their target mRNAs, often overlapping the ribosome binding region [9]. Deletions of sRNA genes have been made in several species. Often, no phenotypic difference to the wild-type could be observed, or the phenotype was very mild. Therefore, it has been concluded that in bacteria sRNAs act as an additional layer on top of other regulatory networks, and that they have evolved to fine-tune the regulation of gene expression [10], [11].

In eukaryotes, the best-studied classes of sRNAs are miRNAs, siRNAs, and piRNAs [12]–[16]. All of them are about 20 nt, much shorter than bacterial sRNAs. miRNAs are typically involved in translational regulation. However, in contrast to bacterial sRNAs, eukaryotic miRNAs repress translation of their target mRNAs by binding to the 3′-UTR. In addition, their malfunction often has dramatic consequences. Mutations in miRNAs of *Caenorhabditis elegans* can cause severe defects in development [17], [18]. In humans miRNA dysfunction can cause severe diseases, e.g. multiple sclerosis, Parkinson's disease, and Alzheimer's disease. Furthermore, miRNA dysfunction has been associated with mental retardation and with various kinds of cancer [14], [16], [19].

The first class of sRNAs discovered in the third domain of life, the archaea, were snoRNAs, which are involved in posttranscriptional modification of stable RNAs, i.e. base methylation and pseudouridine formation. Based on their conserved biochemical role they were named snoRNAs in spite of the lack of a nucleus and nucleolus in archaea ([20], [21] for a review see [22]).

About ten years ago bioinformatic predictions and experimental RNomics led to the discovery of additional sRNAs in *Archaeoglobus fulgidus*, *Methanocaldococcus janaschii*, *Pyrococcus furiosus*, and *Sulfolobus solfatoricus*, indicating that sRNAs are also widespread in archaea [23]–[26]. Recently high throughput sequencing approaches resulted in a global analysis of the sRNA inventory of three archaeal species, *Methanosarcina mazei*, *S. solfataricus*, and *Haloferax volcanii* [27]–[29]. In the three species about 200 intergenic and antisense sRNAs were detected, showing that the numbers of sRNAs in archaea is similar to that in bacteria. Archaeal sRNAs are about 50–400 nt in size, similar to bacterial sRNAs. Apart from snoRNAs and very recently identified CRISPRs, in depth functional studies about archaeal sRNAs are available only for two species, *M. mazei* and *H. volcanii*. Studies with *M. mazei* have concentrated on nitrogen metabolism and revealed nitrogen source-dependent differential expression of various sRNA genes. In addition, a sRNA gene deletion mutant had a growth defect under nitrogen limiting conditions, and one sRNA was found to have two target mRNAs [4], [27], [30], [31].

In *H. volcanii* sRNAs have been identified using RNomics, bioinformatic predictions in combination with experimental verification, and high throughput sequencing [29], [32]–[35]. Differential expression of sRNA genes under various conditions was verified using Northern blot analysis, microarray analysis, and high throughput sequencing (HTS). The first two sRNA mutants that were constructed exhibited severe phenotypes at the maximal growth temperature and a very low salt concentration, respectively [33]. Thus it appeared that lack of an haloarchaeal sRNA can have a larger impact on the phenotype of the cell than lack of a typical bacterial sRNA and that construction and analysis of mutants might be a promising strategy to unravel the biological roles of archaeal sRNAs. Therefore, the current study focused on the generation of a set of sRNA gene deletion mutants and their phenotypic characterization under various conditions. In addition, the transcriptomes of a sRNA gene deletion mutant and the parent strain were compared. Furthermore, the conservation of the analyzed sRNAs is discussed.

## Results

### Generation of sRNA deletion mutants in *Haloferax volcanii*

145 intergenic sRNAs had been identified using RNomcis, high throughput sequencing, and two bioinformatic approaches [29], [32]. sRNA genes were selected as candidates for the generation of deletion mutants based on the following criteria: 1) neighbouring genes with known functions involved in biologically “interesting” processes, 2) a distance of at least 40 bp between sRNA gene and neighbouring genes, because the average length of 3′-UTRs in haloarchaea is 40 nt and it should be avoided to delete conserved motifs in UTRs instead of *bona fide* sRNAs, and 3) differential regulation, which was indicated by the results of high throughput sequencing that had been performed using RNAs from cultures grown under three different conditions to exponential phase and stationary phase, respectively [29].

Successful mutant construction was verified by Southern blot analysis. Three examples are shown in Figure S1, which includes schematic overviews of the genomes and Southern blot results of the parent strain, a Pop-In variant, and the deletion mutant. In total 27 sRNA gene deletion mutants were generated, and all were characterized by Southern Blot analysis (data not shown). Table 1 gives an overview of sRNA genes, their genomic localizations, sRNA sizes, and the method of detection.

**Table 1:** Overview of sRNA gene deletion mutants and selected features.

sRNA	parent strain	HVO-no.	genomic localisation	size [nt]	RNomics	bioinf. predictions	HTS	phenotypic screening
**sRNA_63_**	H26	HVO_B0326s	pHV3 (378513-690/R)	178	**yes**	no	no	**yes**
**sRNA_132_**	H26	HVO_1405s	CHR (1280196-333/R)	138	**yes**	no	**yes**	**yes**
**sRNA_168_**	H26	HVO_A0133s	pHV4 (131765-909/R)	145	**yes**	no	no	**yes**
**sRNA_194_**	H26	HVO_2011s2	CHR (1857632-748/R)	117	**yes**	no	**yes**	**yes**
**sRNA_235_**	H26	HVO_2284s2	CHR (2148186-335/R)	200	**yes**	no	**yes**	**yes**
**sRNA_288_**	H26	HVO_B0116s	pHV3 (130470-618/F)	141	**yes**	no	no	**yes**
**sRNA_308_**	H26	HVO_1355s	CHR (1234567-716/R)	151	**yes**	no	**yes**	**yes**
**sRNA_362_**	H119	HVO_A0137s	pHV4 (135199-340/F)	143	**yes**	no	no	**yes**
**sRNA_450_**	H119	HVO_A0242s	pHV4 (246414-573/F)	160	**yes**	no	no	**yes**
**sRNA_479_**	H119	HVO_A0204s2	pHV4 (207708-873/F)	166	**yes**	no	no	**yes**
**sRNA_500_**	H26/H119	HVO_1187s	CHR (1080188-342/F)	155	**yes**	**yes**	**yes**	**yes**
**sRNA_529_**	H119	HVO_A0126s	pHV4 (124597-744/F)	149	**yes**	no	**yes**	**yes**
**sRNA_H225.2R_**	H26	HVO_1727s2	CHR (1598008-103/F)	96	no	**yes**	no	**yes**
**sRNA_htsf182_**	H26	HVO_1456s	CHR (1329307-323/F)	17	no	no	**yes**	**yes**
**sRNA_htsf209_**	H26	HVO_1724s	CHR (1594231-47/F)	17	no	no	**yes**	**yes**
**sRNA_htsf242_**	H26	HVO_1933s	CHR (1781298-327/R)	30	no	**yes**	**yes**	**yes**
**sRNA_htsf339_**	H26	HVO_2399s	CHR (2268672-88/F)	17	no	no	**yes**	**yes**
**sRNA_hts359_**	H26	HVO_2520s	CHR (2386663-81/R)	19	no	no	**yes**	**yes**
**sRNA_htsf416_**	H26	HVO_2834s	CHR (2670126-42/F)	17	no	**yes**	**yes**	**yes**
**sRNA_htsf467_**	H26	HVO_C0057s	pHV1 (57795-813/F)	19	no	no	**yes**	**yes**
**sRNA_htsf468_**	H26	HVO_C0057s2	pHV1 (58689-881/F)	193	no	no	**yes**	**yes**
**sRNA_htsf494_**	H26	HVO_B0234s	pHV3 (278591-694/R)	104	no	no	**yes**	**yes**
**sRNA_htsf574_**	H26	HVO_A0638s	pHV4 (635713-733/R)	21	no	no	**yes**	**yes**
* **sRNA_hts4_**	H119	HVO_2019s	CHR (1865357-541/F)	185	no	no	**yes**	**yes**
* **sRNA_hts10_**	H119	HVO_2213s	CHR (2078656-816/F)	161	no	**yes**	**yes**	**yes**
**sRNA_hts14_**	H119	HVO_0104s	CHR (102869-3017/F)	149	no	no	**yes**	**yes**
* **sRNA_hts21_**	H119	HVO_2583s	CHR (2436224-377/F)	154	no	no	**yes**	**yes**

* published in Heyer *et al.*, 2012.

### Phenotyping of *H. volcanii* sRNA gene deletion mutants

The phenotypes of the 27 deletion mutants were compared with that of the respective parent strain under ten different conditions. Cultures were grown in synthetic medium on four different carbon sources, i.e. casamino acids, glucose, xylose, and acetate. Thereby very different growth rates were tested. Cultures were also grown at a low salt concentration of 1.2 M NaCl and a high concentration of 4.0 M NaCl in addition to the optimal NaCl concentration of 2.1 M. In addition, four different stresses were applied, i.e. a temperature-downshift, an osmotic down-shift, oxidative stress, and ethanol stress. The growth curves were used to compare three parameters between mutants and parent strains, i.e. the doubling time during exponential growth, the growth yield in stationary phase, and the lag phase before the onset of growth (the latter only qualitatively). All assays were performed in three independent biological replicates and thus about 1000 growth curves were recorded. This phenotyping approach was only possible because growth of *H. volcanii* in microtiter plates has recently been established [36]. Average values and standard deviations were calculated and tabulated. All growth yields and doubling times are summarized in Tables S1 and S2. For a better overview Table 2 summarizes only those mutants and conditions under which a difference between mutant and parent strain was observed. Only deviations of at least 10% were included. All three readouts of the growth curves (lag phase, growth rate, growth yield) are included even if the respective mutant deviates in only one of them. Therefore, the values with deviation between mutant and parent strain are shown in bold.

**Table 2:** Phenotypic differences between sRNA mutants and parent strain.

sRNA deletion mutant	condition	lag-phase	doubling time [% of wild type] (time in hours)	growth yield [% wild type]	swarming [% of wild type]
**H26Δ63**	cas	**elongated**	108% (6.0)	97%	**121%**
	xylose	**shortened**	**72% (10.5)**	**110%**	
	glucose (4 M)	equal	99% (6.4)	**85%**	
**H26Δ132**	4 mM paraquat		**71% (8.0)**	**125%**	83%
**H26Δ168**	glucose (1.2 M)	equal	**116% (10.8)**	98%	**40%**
**H26Δ194**	cas	**shortened**	108% (5.9)	102%	79%
	glucose (1.2 M)	**shortened**	**89% (8.3)**	101%	
	glucose (2.1 M)	**shortened**	93% (6.6)	91%	
	glucose (4 M)	**shortened**	**81% (5.3)**	**87%**	
**H26Δ235**	glucose (1.2 M)	**elongated**	105% (9.7)	107%	77%
	glucose (2.1 M)	**elongated**	95% (6.8)	94%	
	glucose (4 M)	equal	103% (6.7)	**70%**	
**H26Δ288**	glucose (4 M)	equal	98% (6.4)	**77%**	85%
**H26Δ308**	4 mM paraquat		95% (10.6)	**126%**	**35%**
**H119Δ362**	cas	**elongated**	**124% (7.8)**	98%	n.d.
	xylose	**elongated**	n.e.	92%	
	acetate	**elongated**	**much longer**	**72%**	
	glucose (2.1 M)	**elongated**	109% (7.9)	91%	
	glucose (4 M)	**elongated**	95% (6.1)	92%	
**H119Δ450**	acetate	**elongated**	97% (16.5)	90%	n.d.
	glucose (1.2 M)	equal	**57% (10.4)**	**110%**	
**H119Δ479**	acetate	**elongated**	**139% (23.6)**	90%	n.d.
	glucose (1.2 M)	equal	**55% (10.0)**	**114%**	
**H26Δ500**	4 mM paraquat		94% (10.5)	**129%**	**29%**
**H119Δ529**	glucose (4 M)	equal	**118% (7.6)**	**87%**	n.d.
**H26ΔH225.2R**	acetate	**elongated**	**much longer**	98%	94%
	glucose (1.2 M)	**elongated**	**172% (19.1)**	**77%**	
	glucose (2.1 M)	**elongated**	92% (6.9)	**88%**	
	salt stress		93% (9.0)	**137%**	
	cold stress		equal	**123%**	
**H26Δhtsf_182**					**63%**
**H26Δhtsf_242**	glucose (2.1 M)	**elongated**	108% (9.1)	101%	n.d.
**H26Δhtsf_339**	acetate	**elongated**	102% (19.8)	101%	n.d.
**H26Δhtsf_416**	acetate	**elongated**	93% (18.0)	107%	70%
	glucose (2.1 M)	**elongated**	**110% (9.3)**	98%	
**H26Δhtsf_468**	xylose	equal	**35% (24.9)**	113%	**47%**
	glucose (2.1 M)	equal	**140% (10.6)**	**63%**	
	glucose (4 M)	**elongated**	**120% (5.9)**	**78%**	
**H26Δhtsf_494**	acetate	equal	**82% (15.8)**	107%	72%
**H26Δhtsf_574**	glucose (2.1 M)	**elongated**	**117% (9.9)**	101%	n.d.
**H119Δhts4** *	acetate	**elongated**	n.e.	**76%**	n.d.
	glucose (2.1 M)	equal	106% (7.7)	**87%**	
	glucose (4 M)	n.e.	110% (7.0)	**84%**	
**H119Δhts10** *	acetate	**elongated**	n.e.	**87%**	n.d.
	glucose (1.2 M)	equal	81% (14.7)^1^	**113%**	
	glucose (4 M)	n.e.	106% (6.8)	**89%**	
**H119Δhts14**	xylose	n.e.	**83% (72.4)**	**121%**	113%
	acetate	**shortened**	**85% (28.4)**	**138%**	
	glucose (2.1 M)	n.e.	98% (7.5)	**113%**	
	salt stress		**68% (11.7)**	**203%**	
	cold stress		98% (8.4)	**74%**	
**H119Δhts21** *	acetate	**shortened**	**86% (28.7)**	**134%**	100%
	glucose (2.1 M)	n.e.	102% (7.8)	**120%**	
	salt stress		**64% (11.0)**	**264%**	

1 Iniquitous growth of the wild type.

* published in Heyer et al., 2012.

In addition to the growth experiments described above cultures were also grown in complex medium and cell morphology was observed microscopically during exponential growth phase and at stationary phase. Furthermore, swarm plate assays were also performed to test swimming velocity and chemotaxis. The results of these assays are described below.

24 of the 27 deletion mutants exhibited a phenotypical difference from the parent strain under at least one of the tested conditions, while the remaining 3 deletion mutants behaved identically to the parent strain in all assays. In the following paragraphs specific aspects of the observed differences are discussed.

### sRNA gene deletion mutants and metabolic regulation

**Figure 1:**
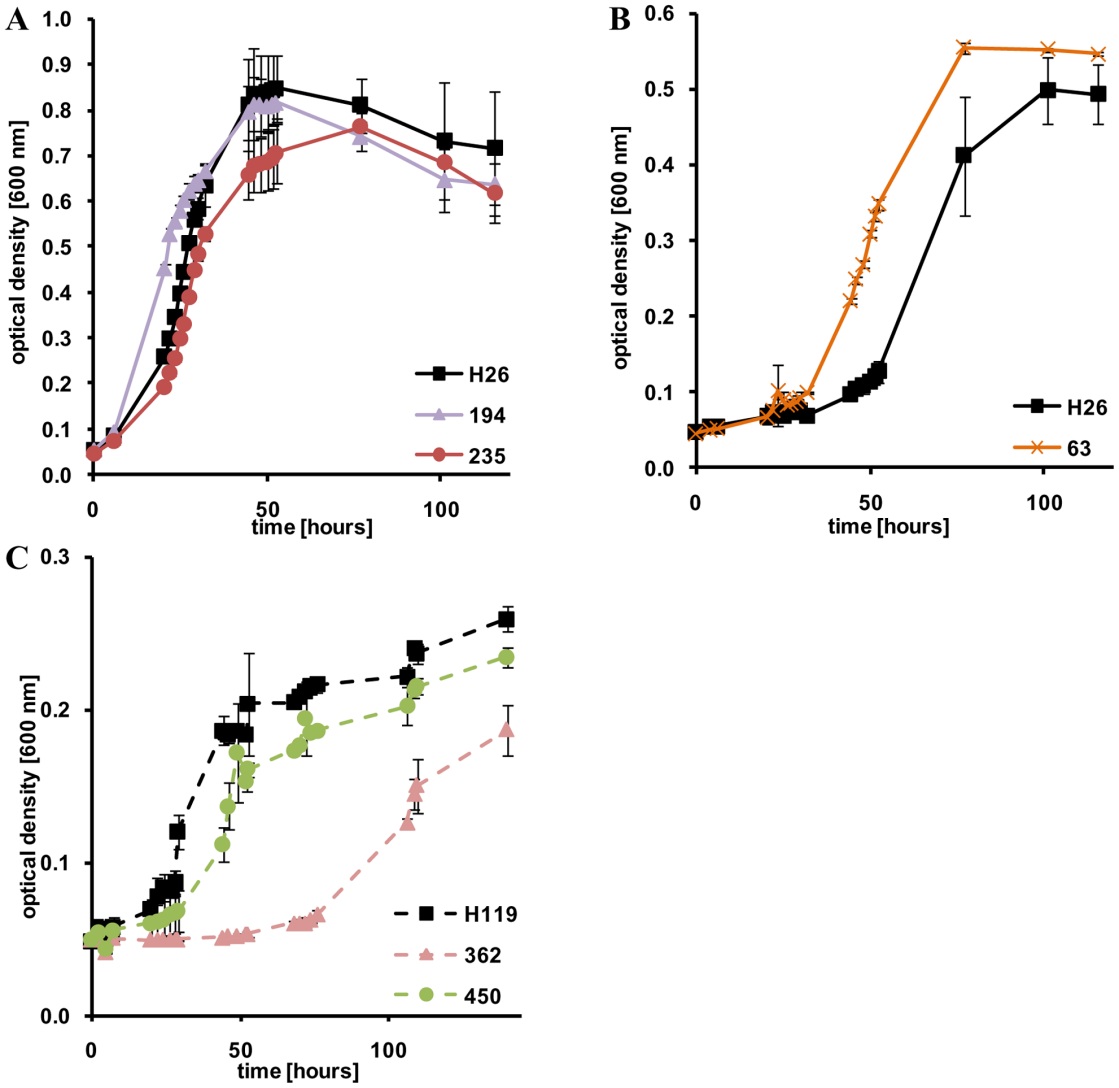
Growth curves of the parent strain (black) and selected mutants (in color) grown with different carbon sources. **A.** Parent strain, Δ194 (purple), and Δ235 (red) grown on glucose. **B.** Parent strain and Δ63 (orange) grown on xylose. **C.** Parent strain, Δ450 (green), and Δ362 (rose) grown on acetate.

Growth on four different carbon sources was tested to reveal whether haloarchaeal sRNAs are involved in metabolic regulation. The preferred carbon source of *H. volcanii* is casamino acids, which result in doubling times of about 4 hours in Erlenmeyer flasks and about 5.5 hours in microtiter plates. The doubling times in microtiter plates are 7–8 hours for glucose, about 15 hours for xylose and about 18 hours for acetate. Table 3 shows that there is a clear tendency that sRNAs are more important for regulation at lower growth rates. On the preferred carbon source casamino acids only one mutant has a different doubling time than the parent strain (24% longer), and the growth yields of all 27 deletion mutants were indistinguishable from that of the parent strain. In contrast, on the poorest carbon source, acetate, 6 mutants had a different doubling time and 5 mutants had a different growth yield than the parent strain. In addition, the phenotypic differences were much milder for growth on casamino acids, glucose (Fig. 1A), and xylose (Fig. 1B) than for growth on acetate (Fig. 1C). Some phenotypic deficiencies on acetate were quite dramatic, for example deletion mutant Δ362 had a lag phase of 80 hours before the onset of growth and a 40% reduced growth yield (Fig. 1C). It should be noted that a null phenotype was not observed for any of the 27 mutants on any of the four carbon sources.

**Table 3:** Numbers of mutants with phenotypic difference to the parent strain during growth on four different carbon sources.

carbon source	lag phase	doubling time	growth yield
casamino acids	3	1	0
glucose	7	3	5
xylose	2	3	2
acetate	10	6	5

Taken together, the phenotypic differences to the parent strain were very diverse on the different carbon sources. On the best carbon source, casamino acids, the phenotypic differences were small, while on the worst carbon source, acetate, many mutants had growth defects, including the most severe loss of function phenotype observed at all.

### Growth of sRNA gene deletion mutants under extreme salt concentrations

**Figure 2:**
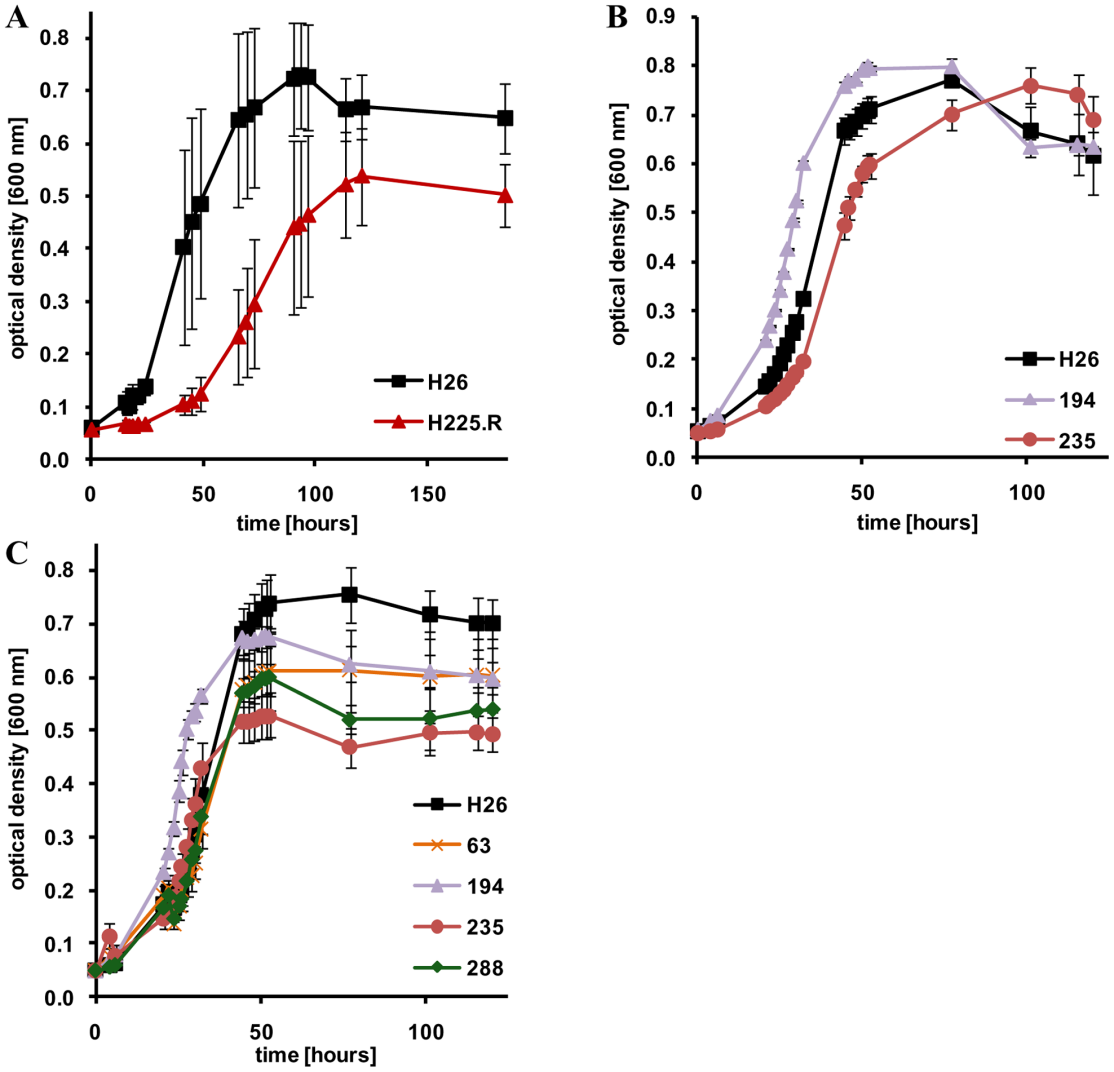
Growth curves of the parent strain (black) and selected mutants (in color) grown at extreme salt concentrations. **A.** Parent strain and ΔH225.2R (red) grown at 1.2 M NaCl. **B.** Parent strain, Δ194 (purple), and Δ235 (red) grown at 2.1 M NaCl. **C.** Parent strain, Δ63 (orange), Δ194 (purple), Δ235 (red), and Δ288 (green) grown at 4.0 M NaCl.

One of the first two *H. volcanii* sRNA gene deletion mutants that had been generated previously was important for growth at low salt concentrations [33]. Therefore, growth of the mutants at a very low NaCl concentration of 1.2 M NaCl were compared to that of the parent strain. In addition, also growth at the optimal concentration of 2.1 M NaCl and a very high concentration of 4.0 M NaCl were tested. Table 4 shows that phenotypic differences were observed at all three NaCl concentrations and there was no clear accumulation of mutants with phenotypes at any of the three salt concentrations. Fig. 2 shows selected growth curves to exemplify that phenotypic differences were observed for the lag phase, the growth rate and/or the growth yield. Fig. 2A shows growth of a mutant that had a lower growth rate and a lower growth yield (ΔH225.2R), while Fig. 2B shows two mutants that have the same growth rate and growth yield as the parent strain, but a slightly shorter or larger lag phase, respectively. Fig. 2C shows three mutants that initially grew indistinguishably from the parent strain but had a reduced growth yield (Δ63, Δ235, and Δ288) and also includes deletion mutant Δ194 that had a shorter lag phase than the parent strain under four different conditions (Fig. 1A, Fig. 2B,C, data not shown). In most cases the differences to the parent strain during growth at all three salt concentrations were not very large, and no null phenotype was observed. The biggest differences were observed at 1.2 M NaCl, i.e. a 45% decrease in doubling time (Δ479) and a 72% increase in doubling time (ΔH225.2R). Gain of function phenotypes occurred at all three salt concentrations.

**Table 4:** Numbers of mutants with phenotypic difference to the parent strain during growth at three different NaCl concentrations.

concentration	lag phase	doubling time	growth yield
1.2 M NaCl	4	5	4
2.1 M NaCl	6	3	5
4.0 M NaCl	3	3	8

In total 18 of the 27 deletion mutants had a phenotype at at least one of the three salt concentrations. However, when the phenotype was observed only at 2.1 M NaCl or when the same phenotype was observed at all three salt concentrations it is not clear whether the sRNA is involved in osmotic adaptation or in the regulation of glucose metabolism. This leaves 9 sRNAs that are clearly involved in osmoadaptation (Table 2). Taken together, the results revealed that a considerable fraction of the 27 characterized sRNAs are important for osmoadaptation, while none is essential for growth at very low or high NaCl concentrations

### sRNA deletion mutants and stress adaptation

Many bacterial sRNAs have been found to be involved in stress adaptation [5]. Therefore, *H. volcanii* was exposed to four different stress conditions to reveal whether this might also be true for haloarchaeal sRNAs, i.e. a temperature down-shift, an osmotic down-shift, oxidative stress, and solvent stress.

**Figure 3:**
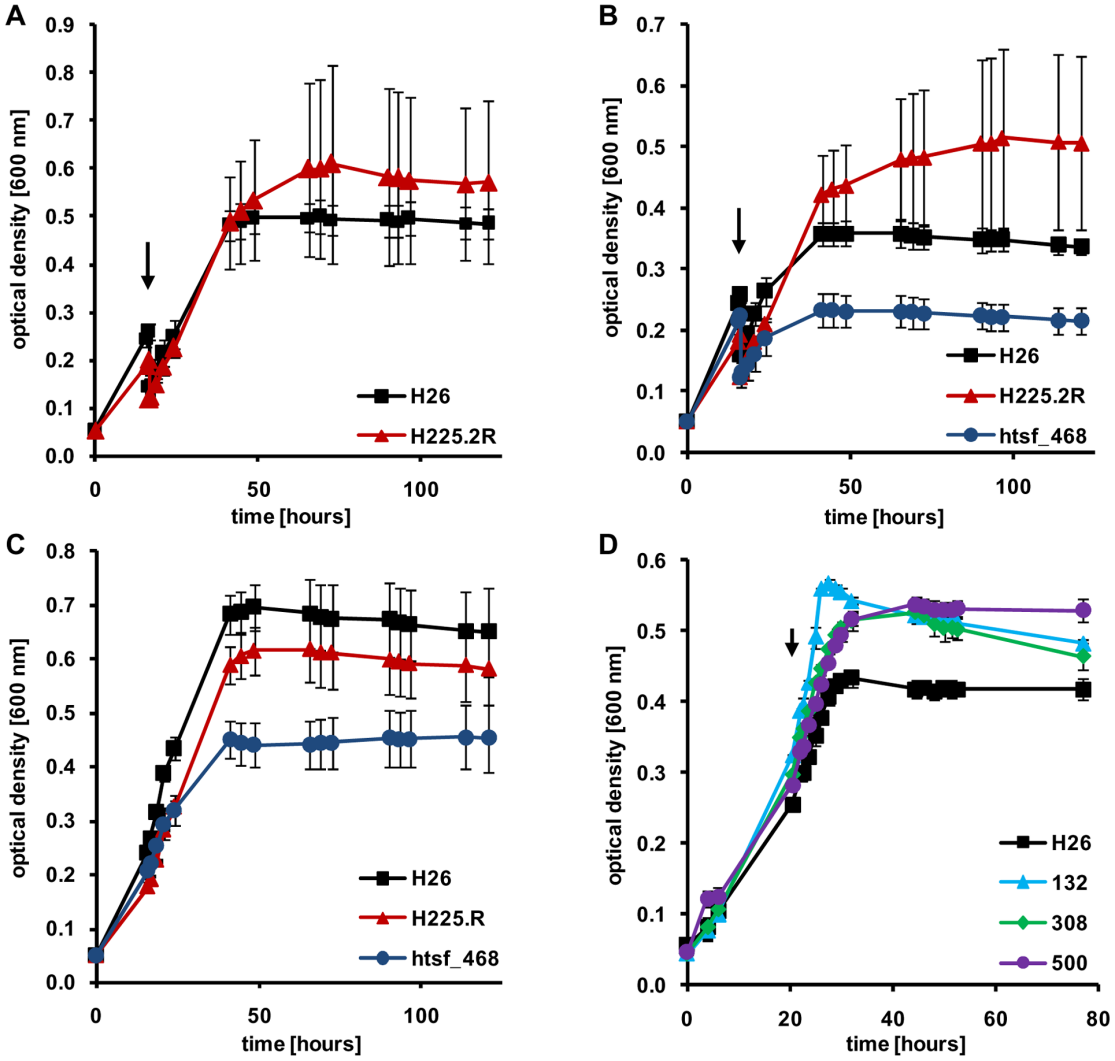
Growth curves of the parent strain (black) and selected mutants (in color) exposed to different stress conditions. **A.** Parent strain (blue) and ΔH225.2P (red) after the application of a temperature down-shift (arrow). **B.** Parent strain, ΔH225.2P (red), and Δhtsf468 (blue) after the application of a osmotic down-shift (arrow). **C.** The same strains as in B. grown in the absence of stress. **D.** Parent strain, Δ132 (blue), Δ308 (green), and Δ500 (purple) after the exposure to oxidative stress (arrow).

The temperature down-shift did not alter the growth rate of any of the 27 deletion mutants compared to the parent strain, and only 2 mutants had a different growth yield. Remarkably, one of the two mutants showed a slightly enhanced growth yield (Fig. 3A). Thus the absence of only one of the 27 sRNAs led to a slight disadvantage after a temperature down-shift (a 26% reduction in growth yield of mutant Δhts14).

Only 3 mutants differed in growth yield from the parent strain after an osmotic down-shift, and one had a slightly different growth rate. Mutant H225.2R exhibited a higher growth yield after the down-shift both in temperature and in salt concentration (Fig. 3A,B), but had a lower growth yield after growth under optimal conditions (Fig. 3C). Therefore, this sRNA does not help in stress adaptation, but, in contrast, its presence is important for optimal growth and it compromises adaptation to at least two different stresses. Also the other two deletion mutants had a higher growth yield than the parent strain after an osmotic down-shift (Δhts14: 203%, Δhts21: 264%).

A few mutants had a lower growth yield than the parent strain after an osmotic down-shift, e.g. Δhtsf468 (Fig. 3B). However, deletion mutant Δhtsf468 (Fig. 3C) and the other mutants had lower growth yields also after growth under optimal conditions, and thus these differences were not stress-related, but carbon source related, and these examples are not included in Table 2. Therefore, none of the 27 sRNAs seems to be important for the adaptation to a sudden osmotic down-shift.

After application of an oxidative stress only 3 mutants had a different growth yield compared to the parent strain, and in all three cases the growth yields of the mutants were higher (Fig. 3D), indicating that the absence of the respective sRNAs enhances rather than compromises the adaptation to oxidative stress. Two additional mutants had a lower growth yield, as expected for a mutant missing a sRNA involved in stress adaptation. However, they also had a lower growth yield after growth under optimal conditions and they were not included in Table 2 as defective in oxidative stress response. Therefore, none of the 27 sRNAs seems to be important for the adaptation to oxidative stress.

The addition of ethanol did not induce a phenotypic difference to the parent strain in any of the 27 mutants.

In summary, only one of the 27 deletion mutant exhibited a slight disadvantage compared to the parent strain after application of one of the four stress conditions and had a slight reduction in growth yield after a temperature down-shift. Therefore, sRNAs do not seem to be very important for stress adaptation in *H. volcanii*. Unexpectedly, 4 of the 27 mutants reached higher growth yields after stress application, indicating that the presence of the respective sRNAs is suboptimal for stress adaptation.

### Characterization of cell morphology

**Figure 4:**
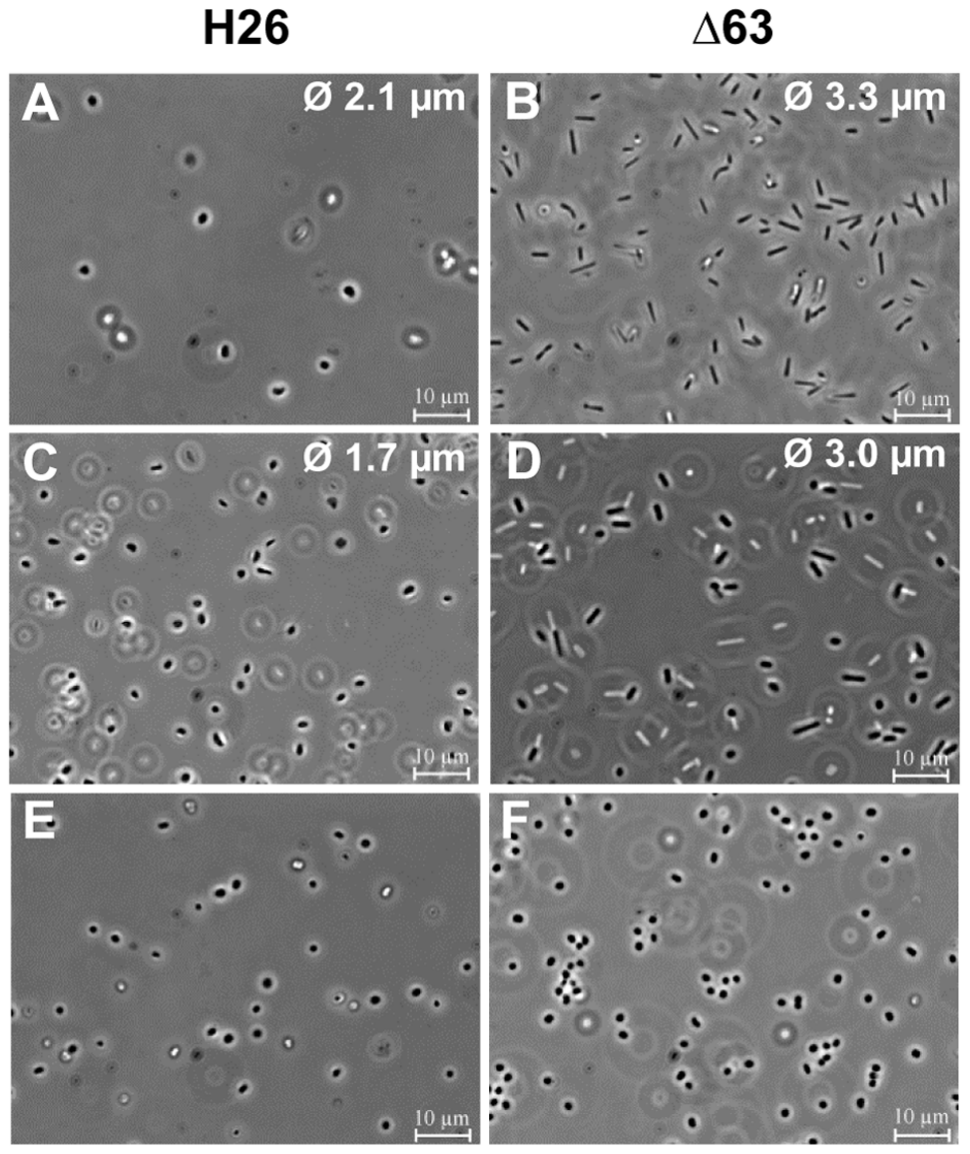
Microscopic pictures of the parent strain and deletion mutant Δ63. Average cell sizes are indicated, and a scale bare is included. **A+B.** Exponential growth phase in complex medium. **C+D.** Stationary phase in complex medium. **E+F.** Exponential phase in synthetic medium with glucose as carbon source.

All mutants were also inspected microscopically throughout the growth curve. Cell morphologies of all but one mutant were indistinguishable from that of the parent strain. In contrast, the average length of mutant Δ63 clearly exceeded that of the parent strain by more than 50%. This was true both for exponential phase (Fig. 4A+B) as well as in stationary phase (Fig. 4C+D). However, this morphological difference was confined to growth in complex medium and was not observed in synthetic media, e.g. growth in synthetic medium on glucose (Fig. 4E+F).

### Comparison of the transcriptomes of mutant Δ63 and parent strain

**Figure 5:**
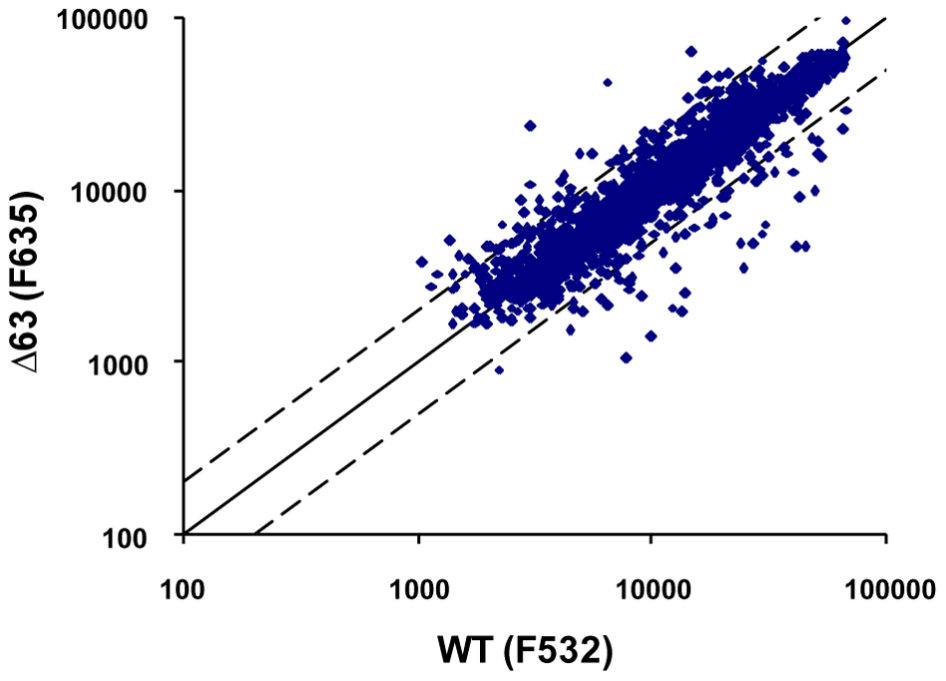
Scatter plot of the microarray analysis of the transcriptomes of parent strain and deletion mutant Δ63. Average signals of four biological replicates are shown. The experiment included a dye swap. The RNA was isolated after the exposures of the cultures to 1% (v/v) ethanol for 15 minutes. The solid line represents the diagonal (identical transcript levels in parent strain and mutant). The dotted lines represent a twofold difference between parent strain and mutant.

None of the 27 sRNA gene deletion mutants differed from the parent strain after the application of a solvent stress with ethanol. However, reporter gene assays with several sRNA promoters had revealed that the promoter of sRNA_63_ was more than tenfold up-regulated after the addition of ethanol (unpublished data). This result indicated that sRNA_63_ might have a function under this condition even if the deletion mutant did not exhibit a phenotype. To analyze a putative role of sRNA_63_ in the presence of ethanol, the transcriptomes of deletion mutant and parent strain were compared after an ethanol shock. A scatter plot of average signals of four biological replicates (including a dye swap) is shown in Fig. 5. The vast majority of transcripts had identical levels in the deletion mutant and the parent strain, but the absence of sRNA_63_ resulted in different levels of several transcripts. In most cases the difference was between two- to threefold and thus not really high (Tables S3 and S4). The highest difference was found for the transcripts of the genes encoding flagellins A1 and A2 (HVO_1210, HVO_1211), which were 16fold up-regulated in the deletion mutant. A slight up-regulation (2.3fold) was also observed for the nearby and functionally related genes of the chemotaxis gene cluster (HVO_1203 - HVO_1207).

**Figure 6:**
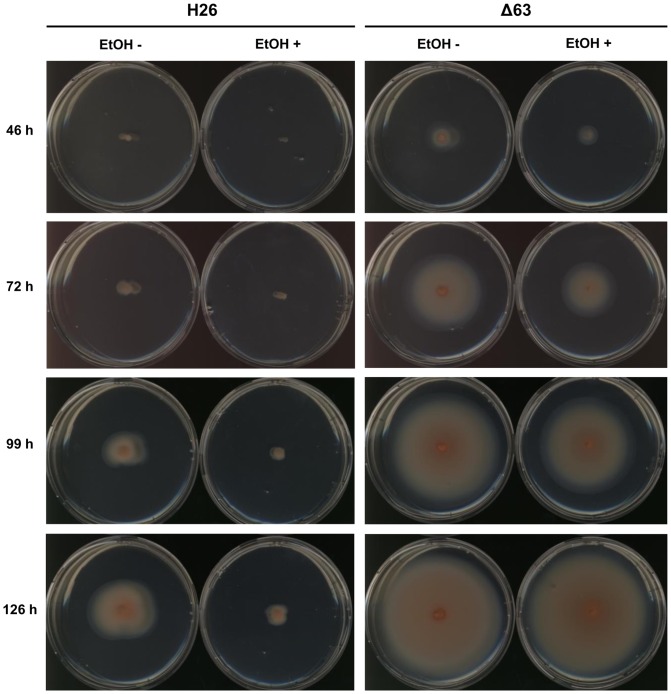
Swarm plate analysis of parent strain (H26) and deletion mutant Δ63 in the absence and presence of 1% (v/v) ethanol, as indicated. The pictures were taken at four different times after inoculation, as indicated.

These results indicated that sRNA_63_ might be involved in the negative regulation of motility and chemotaxis. To test this hypothesis, swarm plate assays were performed for the deletion mutant and the parent strain in the absence and presence of ethanol. Fig. 6 shows that indeed mutant Δ63 had a gain of function phenotype and swarming was considerably reduced in the parent strain containing sRNA_63_. In addition, the presence of ethanol inhibited swarming much more in the parent strain than in the Δ63 mutant, probably mediated via the more than tenfold induction of sRNA_63_ gene expression following ethanol addition.

The transcriptome comparison led to the identification of an additional transcript with a considerably different level in mutant and parent strain, i.e. the transcript of of the gene *muc19* (HVO_2160). The gene encodes an extremely large protein of more than 2200 amino acids. Its serine and threonine content is 24% and thus it can be predicted that it has a high water-binding capacity. It is tempting to speculate that external mucin could shield *H. volcanii* under conditions of low water activity, including organic solvent stress, but experimental evidence is currently not available.

Taken together, the transcriptomes of deletion mutant and parent strain were compared when the strains had no phenotypic difference. This led to the identification of transcripts that are (directly or indirectly) regulated by sRNA_63_. A hypothesis about a biological function was generated, which could indeed be verified using swarm plate assays.

### sRNAs involved in the regulation of behaviour

**Figure 7:**
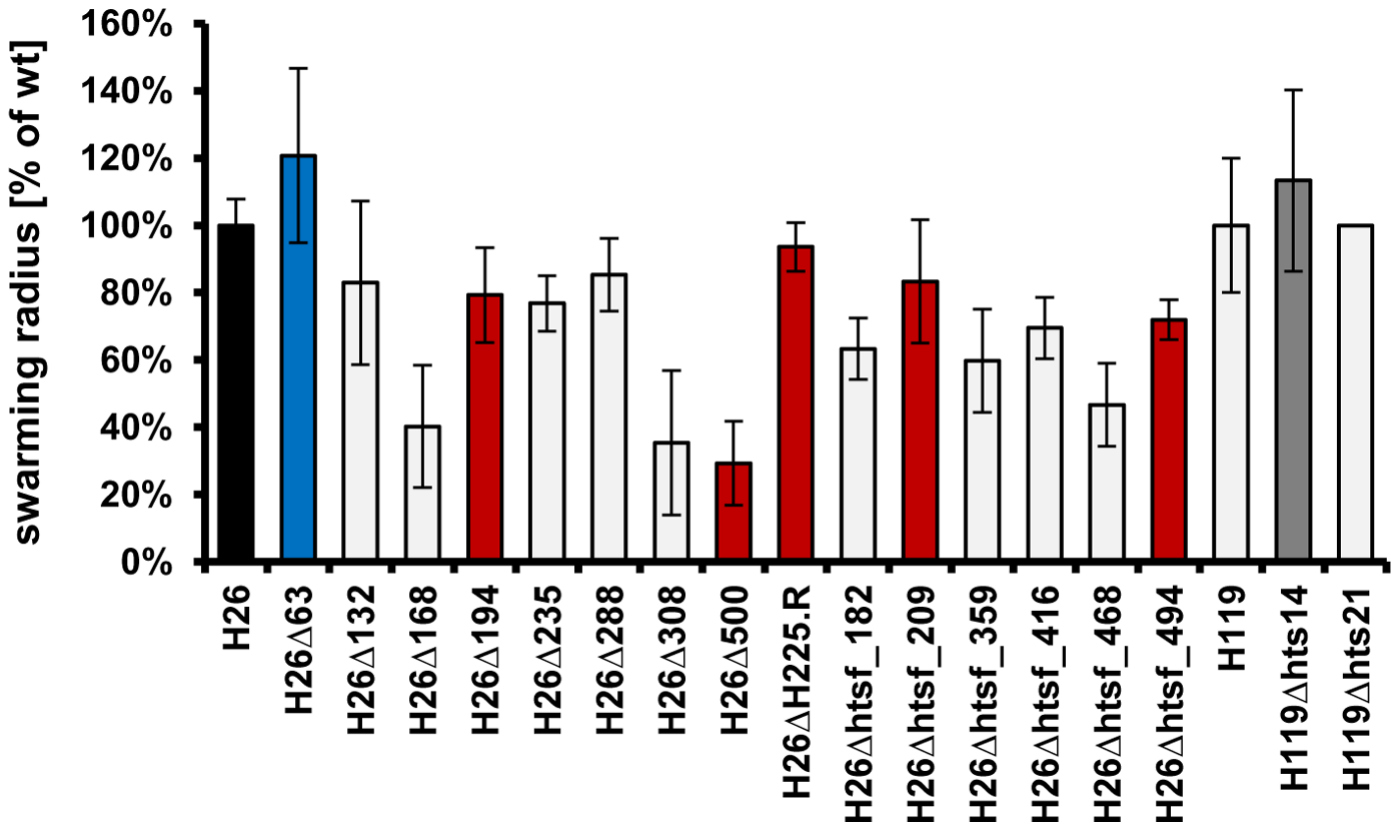
Swarming velocity of the parent strains (black, grey) and 17 mutants. The mutant with a gain of function phenotype is shown in blue, the five mutants with a loss of function phenotype are shown in red. Average values of three replicates and their standard deviations are shown.

The observation that mutant Δ63 and parent strain differed in motility and/or chemotaxis induced the analysis of further sRNA deletion mutants using the swarm plate assay. 16 additional deletion mutants were selected that were in culture at that time, and compared to mutant Δ63 and the parent strain in triplicate swarm plate assays. Average results and their standard deviation are shown in Fig. 7. Of the 17 mutants that were analyzed only mutant Δ63 had a gain of function phenotype and swarmed faster than the parent strain. However, 5 of the mutants showed a loss of function phenotype and swarmed considerably slower than the parent strain. As both motility and chemotaxis are required for swarming, further analyses are required to unravel whether the former or the latter (or both) is affected in the respective mutants. Similar to all other analyses described above, the null phenotype was not observed in any of the mutants. In summary, 6 of 17 analyzed sRNA gene deletion mutants differed from the parent strain in the swarm plate assay, indicating that many sRNAs are involved in the regulation of behaviour in *H. volcanii*.

### Conservation of *H. volcanii* sRNA genes in other haloarchaea

To unravel the degree of conservation of the sRNA genes of H. volcanii in other haloarchaea, BLAST searches with the sequences of the 27 sRNA genes were performed. The results are summarized in Table 5. Surprizingly, 22 of the 27 sRNAs are confined to H. volcanii and do not have homologues in any other haloarchaeon, and one gene is present in only one additional genome. Only 3 of the 27 sRNA genes are highly conserved in haloarchaea and are present in more than 10 additional genomes, and one sRNA gene is present in seven other haloarchaeal genomes. There was no obvious correlation between the degree of conservation of sRNA genes and the severity of the phenotypes of the respective deletion mutant. For example, deletion of the conserved sRNA_H225.2R_ resulted in a pleiotrophic phenotype, but the deletion mutant of the conserved sRNA_htsf359_ was indistinguishable from the parent strain under all tested conditions. On the other hand, deletion of the non-conserved sRNA_194_ led to a pleiotrophic phenotype, and the most severe phenotype, a lag phase of 80 hours on acetate (Fig. 1C), resulted from deleting the non-conserved sRNA_362_. Taken together, evolutionary conservation and importance of haloarchaeal sRNAs seem to be unrelated.

**Table 5:** Conservation of *H. volcanii* sRNAs.

sRNA	present in haloarchaeal genomes (beside *H. volcanii*)
**63**	0
**132**	0
**168**	1
**194**	0
**235**	0
**288**	0
**308**	0
**362**	0
**450**	0
**479**	0
**500**	0
**529**	0
**H225.2R**	19
**htsf_182**	0
**htsf_209**	0
**htsf_242**	7
**htsf_339**	0
**htsf_359**	12
**htsf_416**	0
**htsf_467**	0
**htsf_468**	0
**htsf_494**	0
**htsf_574**	0
**hts4** *	0
**hts10** *	18
**hts14**	0
**hts21** *	0

* published in Heyer *et al.*, 2012.

## Discussion

### Haloarchaeal sRNAs are involved in metabolic regulation, growth at extreme conditions, and regulation of morphology and behaviour, but much less in stress response

Phenotyping a set of 27 deletion mutants is by far the most comprehensive study about the biological functions of sRNAs in archaea. Only six archaeal sRNA gene deletion mutants have been described prior to this study. In *M. mazei*, a deletion mutant of sRNA_154_ had a severe growth defect under nitrogen-limiting conditions, revealing that this sRNA plays a prominent role in the regulation of the nitrogen fixation pathway [4]. As discussed above, two sRNAs of *H. volcanii* were shown to be essential for growth at high temperature and low salt, respectively [33]. Three additional *H. volcanii* mutants were published in a study that mainly focused on the identification of sRNAs and their regulation by high-throughput sequencing using cells grown under three different conditions to exponential phase and to stationary phase, respectively [29]. These three mutants are also included in Tables S1, S2 and Tables 1, 2, and 5 to enable a comprehensive overview of the phenotypic differences of *H. volcanii* sRNA gene deletion mutants and parent strains (marked by asterisks).

**Figure 8:**
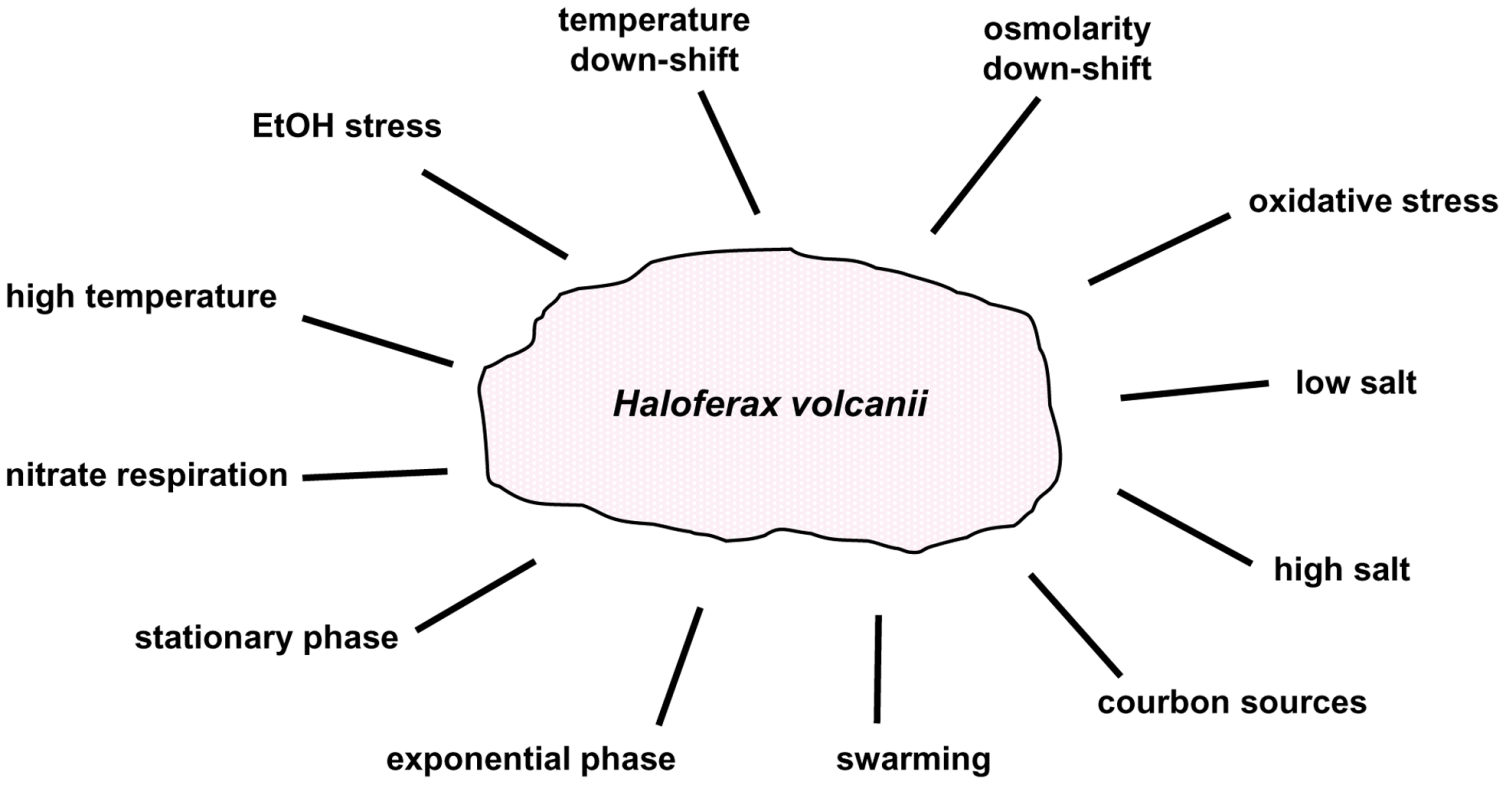
Schematic representation of all conditions under which sRNAs have important functions in *H. volcanii*. The importance of sRNAs was either deduced from phenotypic analysis of deletion mutants (this publication) or from the analyis of differential expression using Northern blot analysis and high throughput sequencing [29], [33].

The fact that 24 of 27 tested sRNA mutants exhibited a phenotype under at least one condition (Table 2) on the one hand showed the potency of the phenotyping approach, and on the other hand it underscored the great importance of sRNAs for haloarchaea. This is in contrast to the importance of sRNAs in bacteria, in which they are believed to be typically responsible for the fine-tuning of gene expression [10], [37]. To our knowledge in no single species the involvement of sRNAs in so many different biological functions has been described as we describe here for the archaeon *H. volcanii*. Fig. 8 gives a schematic overview of the diverse biological functions that haloarchaeal sRNAs are involved in based on experimental evidence presented in this study, i.e. phenotyping of mutants, and in additional studies that analyzed differential expression of sRNA genes [29], [33]. It became obvious that in *H. volcanii* sRNAs are much more involved in the long-term regulation of diverse biological processes than in short-term stress response.

In fact the results of exposing the 27 mutants and the parent strain to four different stress conditions were very surprising and counterintuitive. Only two mutants had very mild loss of function phenotypes, while four mutants had gain of function phenotypes, indicating that sRNAs counteract stress adaptation, at least under the experimental setting in the laboratory.

Unexpectedly it was discovered that 6 sRNAs influence cellular behaviour and their absence can lead both to reduced or to enhanced swarming. Recently it was discovered that also one bacterial sRNA, McaS from *E. coli*, has an influence on motility [38]. It also influences biofilm formation and therefore determines the balance between a sessile and a motile lifestyle of *E. coli* in response to nutrient availability. After the parallel discovery of one archaeal and one bacterial example it is tempting to predict that additional prokaryotic sRNAs will be found to regulate cellular behaviour.

### Many *H. volcanii* sRNA gene deletion mutants have gain of function phenotypes

Very unexpectedly, the deletion of haloarchaeal sRNA genes did not only result in a loss of function phenotype, but also in a gain of function phenotype under at least one condition. In summary, 4 mutants had a shorter lag phase, 9 mutants had a higher growth rate, 10 mutants had a higher growth yield, and one mutant had a higher swarming velocity. As some mutants exhibited several of these phenotypes, the total number of mutants with a gain of function phenotype is much lower than the sum of these values. However, 13 mutants out of the 24 with phenotypic differences to the parent strain exhibited at least one gain of function phenotype under at least one condition, underscoring that this is neither an experimental artefact nor a rare consequence of the absence of a sRNA.

To our knowledge, a gain of function phenotype has as yet not been reported for any sRNA gene deletion mutant of any bacterium. In stark contrast, gain of function phenotypes have been reported as a result of the absence of several miRNAs in higher eukaryotes, and in congruence with that, overexpression of miRNAs has led to loss of function phenotypes [12], [13], [39]. Therefore, it seems that the biological role of sRNAs in haloarchaea and higher eukaryotes is not a one-dimensional optimization of the reaction of the cell to the change in a single parameter. In contrast, their role is to stabilize the system of a cell, organ or organism to the multivariate changes that occur in real life. As haloarchaea are much simpler than higher eukaryotes, they offer the advantage to test under which combination of conditional changes the parent strain is superior to the deletion mutants that exhibit a gain of function phenotype in one-dimensional experiments described above.

### From transcriptomes to phenotypes and functions

Transcriptome analyses using microarrays or high throughput sequencing can be used for the genome-wide parallel analysis of differential levels of sRNAs and mRNAs under various conditions. Comparisons of transcriptomes of cells with different levels of a specific sRNA have the potential to unravel mRNAs that are directly or indirectly regulated by this sRNA. This requires that not only the translational efficiencies, but also the half lives of the transcript are influenced by the sRNA, otherwise translatome analyses would be required. The transcriptome comparison of the deletion mutant Δ63 and its parent strain led to the identification of several transcripts with different levels in the two strains, indicating that sRNAs influence transcript stability in *H. volcanii*, like in *E. coli*. The results of the transcriptome comparison led to the prediction that sRNA_63_ might be involved in the regulation of motility and/or chemotaxis, and this could indeed be verified using a swarm plate assay. This result has induced the swarming analysis of 16 additional deletion mutants, which led to the discovery of 5 additional sRNAs that are involved in the regulation of motility/chemotaxis, which would have otherwise remained unnoticed.

This proof-of-principle experiment indicated that transcriptome comparisons will most probably allow the identification of regulons for additional haloarchaeal sRNAs. This is worth mentioning because bioinformatic target prediction, which has become feasible for bacteria in recent years, is currently not succesful for archaea.

### Evolution of prokaryotic sRNAs

It has been proposed that sRNAs might be reminiscent of an ancient “RNA world” that was and sometimes still is thought to have been crucial for the development of life. Even if that might be true, the current sRNA inventory seems to have evolved very recently. Of the tested sRNAs, the majority is confined to *H. volcanii* alone and is not even present in another species of the same genus, *H. mediterranei*. There are several possible explanations for this observation. One possibility could be that the sRNA genes are confined to plasmids and are not found on the chromosome or that they are predominantly close to transposons, integrated viruses or other mobile genetic elements. In these cases *H. volcanii* would have obtained the sRNA genes by lateral transfer from a species that does not belong to the archaea with sequenced genomes present in the databases. However, the density of sRNA genes (sRNA gene per 100 kbp) is very similar for the three replicons major chromosome, pHV3 and pHV4 and deviates only for the very small replicon pHV1 (Table 6). In addition, despite their names the replicons pHV1, pHV3, and pHV4 are regarded to be chromosomes based on the presence of ORC genes close to origin repeats. There are indeed some sRNA genes close to transposons, but the vast majority is not. Currently it can not be decided between the two alternative possibilities that 1) sRNA genes in *H. volcanii* arose *de novo* during species evolution and 2) sRNA genes were already present in the last common ancestor of the genus *Haloferax* but their sequence evolution was so fast that today it is impossible to detect the homologies by sequence comparisons.

**Table 6:** Distribution of intergenic sRNA genes on the replicons of *H. volcanii*.

Replicon	No. sRNA genes	Size of replicon [kbp]	No. sRNA genes per 100 kbp
Chromosome	167	2847.8	5.86
pHV1	10	85.1	11.75
pHV3	15	437.9	3.43
pHV4	29	635.8	4.56

A very low degree of conservation of sRNAs has also been observed in bacterial species. For example, a bioinformatic screen has led to the identification of 46 sRNAs that are present in *Salmonella* but absent from *Escherichia* [8]. More strikingly, a RNA-seq approach led to the discovery of over 500 intergenic sRNAs in *Pseudomonas aeruginosa*, more than 90% of which had no homologous sequence in any other species [40]. These results underscores the rapid evolution of sRNAs at the species level, and the higher velocity of sRNA evolution compared to the evolution of regulatory proteins might well be an as yet rarely discussed major evolutionary advantage that might explain the wide-spread occurrence of sRNAs in prokaryotes.

### Future aspects

An important point for understanding the molecular mechanism of sRNAs in *H. volcanii* would be the identification of target mRNAs. Genome-wide bioinformatic analyses have been very successfully used for the prediction of targets of bacterial sRNAs. However, attempts to apply programs designed for the prediction of bacterial targets and trained with bacterial sRNA/target mRNA pairs for the prediction of targets for sRNAs of *H. volcanii* failed, indicating that the principles of sRNA function might be different for bacteria and (halo)archaea. Therefore experimental approaches are needed for the identification of haloarchaeal target mRNAs, before haloarchaeal sRNA/target mRNA pairs can be used to guide the optimization of bioinformatic predictions. One approach that is currently pursued is the comparison of the transcriptomes of selected sRNA deletion mutants of the collection described in this contribution with that of the parent strain using DNA microarrays. In each of seven cases analyzed thus far differential transcript levels could be identified. The number of genes varied widely, from less than ten to nearly 100, indicating that sRNAs in *H. volcanii* can have very specific or rather broad regulatory functions, respectively. This is in congruence with the phenotypic analyses of the mutants decribed above, that led to the identification of very specific versus pleiotropic phenotypes, depending on the identify of the mutants. This example should show that the mutants described in this contribution will be valuable tools for many future applications, which aim at understanding regulatory sRNA networks in *H. volcanii*, the molecular mechanisms of sRNA actions, and the signal transduction pathways that lead to the differential production of sRNAs under various environmental conditions.

## Experimental Procedures

### Strains and culture conditions

In this study the two *Haloferax volcanii* strains H26 (Δ*pyrE2*) and H119 (Δ*pyrE2*, Δ*trpA*, Δ*leuB*) were used [41]. H26 has the advantage that it only contains a deletion in the *pyrE* gene and thus is easier to handle than H119. It was used for mutant construction in the Soppa group. H119 has the advantage that it carries two additional deletions in biosynthetic genes that enables double or triple selection schemes to isolate deletion mutants with a severe growth defect. It was used for mutant construction in the Marchfelder group.

In addition, 27 *H. volcanii* H26 or H119 deletion mutants lacking one sRNA gene were analyzed (Table 1). Standard growth conditions for *H. volcanii* were defined as aerobic growth at 42°C and 2.1 M NaCl in synthetic media with glucose as C-source [42].

The *Escherichia coli* strain XL1-blue MRF' (Agilent Technologies, Waldbronn, Germany) was used for cloning and was grown in standard media [43].

### Construction of sRNA deletion mutants

The 27 sRNA genes were deleted using previously described methods [41], [44]. Two different approaches were used, i.e. markerless deletions using strain *H. volcanii* H26 as well as replacement by a selection cassette using strain *H. volcanii* H119.

*Haloferax volcanii* H26 deletion mutants of sRNA genes were generated using the “pop-in/pop-out” method as described previously [41], [44], [45]. In short, two PCR fragments of about 500–600 bp were generated containing either the upstream region or the downstream region of the respective gene (oligonucleotide sequences are available upon request). These PCR fragments had an overlap that enabled the amplification of a “fusion fragment” in a second PCR. The “fusion fragments” containing an internal deletion of the respective sRNA gene were cloned into the vector pMH101 [44]. The resulting plasmid was verified by sequencing and used to transform *H. volcanii* strain H26 [41]. The resulting “pop-in” variant was selected on uracil-free medium. The subsequently selection of the “pop-out” variant was on complex medium containing uracil and 5′-FOA (150 mg/ml). The verification of clones containing the deletion version of the sRNA genes was done by PCR and Southern blot analysis.

The genes for sRNA_194_, sRNA_362_, sRNA_450_, sRNA_479_, sRNA_500_, sRNA_529_ as well as the genes for the Hts sRNAs 1–21 were replaced by marker genes (*trp* or *leuB*) using the pop in-pop-out method described previously [41], [45], [46]. After the amplification of the sRNA genes with additional 500 base pairs up- and downstream the PCR fragment was cloned into the integration vector pTA131 (primers used for amplification are available upon request). To introduce a *Sna*BI restriction site and to delete the entire sRNA gene an inverse PCR was performed on the resulting plasmids. After ligation of the resulting PCR product and digestion with *Sna*BI the *trpA* marker gene was cloned into the plasmids, yielding constructs that contain the up- and downstream region of the sRNA genes and instead of the sRNA gene the *trpA* marker gene (or in the case of the Hts1 sRNA the *leuB* marker gene). To generate pop in-clones *Haloferax* strain H119 was transformed with the plasmids and subsequently pop out-cells were isolated by plating on 5-fluoro-orotic acid (5-FOA) medium. Successful replacement of the sRNA gene was proven by Southern blot hybridisation as described previously [43], [47]. Shortly, chromosomal DNA was isolated from wild type and deletion strains, digested with *Sal*I, separated on 0.8% agarose gels and transferred to a nylon membrane (Hybond™-N, GE Healthcare). As hybridisation probes the up- or downstream regions were amplified and labelled.

### Phenotyping in microtiter plates

For phenotypic comparison, parent strains *H. volcanii* H26 or H119 and the corresponding deletion mutants were grown under different conditions in 96well microtiter plates as described previously [36]. Growth was monitored at 600 nm using a microtiterplate reader (Spectramax 340, Molecular Devices, Sunnyvale, CA, USA).

Fig. S2A gives a schematic overview of the usage of microtiter plates for the phenotyping of sRNA gene deletion mutants, Fig. S2B shows one example. The outmost wells could not be used for culture growth, but were used as “evoporation barriers”, leaving 60 wells per plate for growth experiments. Accordingly a single microtiter plate enabled the analysis of eight sRNA gene deletion mutants, the parent strain and a negative control in triplicate cultures at two different conditions. Accordingly a shaker for six microtiter plates enabled the parallel analysis of eight mutants under 12 different conditions.

The tested growth conditions and stress conditions are listed in Table S1. Three biological replicates were performed and the average values of growth yield, growth rate, the length of lag-phase and their standard deviations were calculated.

### Swarming analysis

To characterize the swarming behaviour, plates containing complex medium with 0.3% (w/v) agar were used. 2 µl of mid-exponential cultures (4×10^8^ cells/ml) of parent strain and deletion mutants, respectively, were added to the middle of the plates. The plates were sealed in a plastic bag, incubated at 42°C and the swarming radii were measured daily.

### Microscopic analysis

The morphologies and sizes of sRNA deletion mutants were compared with the parent strain under different conditions and growth phases using a light microscope (Carl Zeiss, Jena, Germany).

### Isolation of RNA and DNA microarray analysis

For RNA isolation cells were grown to mid exponential growth phase and shocked for 15 minutes with 1% Ethanol. RNA was isolated immediately with the RNA isolation midi kit (Qiagen, Hilden, Germany) according to the manufacturer's instructions. To enable the isolation of RNA molecules smaller than 200 nt, the RW1 buffer was omitted. To exclude a DNA contamination, a DNase treatment was performed while the RNA was bound to the ion exchange column. The RNA concentration was determined photometrically, and the integrity was controlled using an analytical agarose gel electrophoresis.

The isolated control (H26) and sample (Δ63) RNA were reverse transcribed into Cy3-dUTP or Cy5-dUTP labelled cDNA using random hexamer oligonucleotides and M-MLV reverse transcriptase RNase H minus (Promega, Mannheim, Germany). cDNA generation and its preparation for hybridization on a self-constructed DNA microarray for *H. volcanii* were performed in four independent experiments, including a dye swap, as described previously [48]. The analysis of DNA microarray results has been described in a former study [49].

## Supporting Information

Figure S1 **Verification of sRNA gene deletion mutant construction.** Schematic overviews of the genomic organizations of the parent strain (wt), two different possibilities of Pop-In variants (PI-1, PI-2), and the deletion mutant after Pop-Out selection (PO) are shown to the left. The PCR fragments used for the construction of the deletion mutant are shown as boxes (F1, F2). Probes for Southern blot analysis are shown as black bars above the genome. Relevant restriction sites are indicated by arrows and the sizes of hybridizing restriction fragments are shown. The results of Southern blot analyses are shown to the right, the sizes of the hybridizing fragments are indicated. **A.–C.** Three examples for the deletion of sRNA genes are shown.
(PPTX)

Figure S2 **Growth of** ***Hfx. volcanii*** **in microtiter plates.** **A.** Schematic overview of the usage of microtiter plates for the phenotyping of mutants. The blue area indicates wells that were filled with 1 M NaCl as an evaporation barrier. The dotted line separates wells used for cultures exposed to condition 1 and condition 2. c – negative control, wt – parent strain, m1–8 – mutants. **B.** One example of a microtiter plate.
(PPTX)

Table S1 **Summary of the growth yields of 27 sRNA gene deletion mutants and parent strains grown under ten different conditions.** Three biological replicates were performed, and average values and standard deviations were calculated.
(XLS)

Table S2 **Summary of the growth rates of 27 sRNA gene deletion mutants and parent strains grown under ten different conditions.** Three biological replicates were performed, and average values and standard deviations were calculated.
(XLS)

Table S3 **Results of the transcriptome comparison of deletion mutant Δ63 and parent strain: Compilation of genes more than twofold up-regulated in Δ63.**
(XLS)

Table S4 **Results of the transcriptome comparison of deletion mutant Δ63 and parent strain: Compilation of genes more than twofold down-regulated in Δ63.**
(XLS)
